# Repression of TCF3/E2A contributes to Hodgkin lymphomagenesis

**DOI:** 10.18632/oncotarget.9210

**Published:** 2016-05-06

**Authors:** Hanfeng Guan, Linka Xie, Thomas Wirth, Alexey Ushmorov

**Affiliations:** ^1^ Department of Orthopaedic Surgery, Tongji Hospital, Tongji Medical College, Huazhong University of Science and Technology, Wuhan, China; ^2^ Institute of Physiological Chemistry, University of Ulm, Ulm, Germany; ^3^ Cancer Center, Union Hospital, Tongji Medical College, Huazhong University of Science and Technology, Wuhan 430022, China

**Keywords:** classical Hodgkin lymphoma (cHL), TCF3/E2A, cell death

## Abstract

Although Hodgkin and Reed-Sternberg (HRS) cells of classical Hodgkin lymphoma (cHL) derived from germinal or post germinal B cells, they have lost the B cell phenotype in the process of lymphomagenesis. The phenomenon can be at least partially explained by repression of B-cell-specific transcription factors including TCF3, early B-cell factor 1 (EBF1), SPI1/PU.1, and FOXO1, which are down-regulated by genetic and epigenetic mechanisms. The unique phenotype has been suspected to be advantageous for survival of HRS cells. Ectopic expression of some of these transcription factors (EBF1, PU.1, FOXO1) indeed impaired survival of cHL cells. Here we show that forced expression of *TCF3* causes cell death and cell cycle arrest in cHL cell lines. Mechanistically, TCF3 overexpression modulated expression of multiple pro-apoptotic genes including BIK, APAF1, FASLG, BOK, and TNFRSF10A/DR4. We conclude that TCF3 inactivation contributes not only to extinguishing of B cell phenotype but also to cHL oncogenesis.

## INTRODUCTION

Classical Hodgkin lymphoma (cHL) is characterized by the presence of mononucleated Hodgkin cells and multinucleated Reed-Sternberg cells (HRS) surrounded by the overwhelming host cells of “inflammatory infiltrate”. HRS cells are derived from germinal center (GC) B cells which have lost their B cell program in the process of malignant transformation [1]. Multiple pathways that are critical for evading apoptosis and for promotion of cell growth are constitutively activated in HRS cells. They include NF-κB, the Jak-Stat, PI3K-Akt, Erk, AP1, and NOTCH pathways [1]. HRS cells not only have lost their B cell identity, but also acquired expression of markers of other lineages, especially that of T lymphocytes like NOTCH1and GATA3 [1]. Repression of the transcription factors regulating maintenance of B cell program including POU2F2, POU2AF1, SPI1/PU.1, EBF1, and FOXO1 by genetic and epigenetic mechanisms was shown to be responsible for the loss of specific B-cell markers in cHL [1, 2]. Other important B-cell factors are E47 and E12 proteins that are encoded by the *TCF3* gene. TCF3 is expressed in HRS cells, but its function is antagonized by MSC/ABF1, ID2, and NOTCH1 [2, 3]. The reprogramming of HRS cells by the abnormal activities of these lineage regulators has been proposed to be of advantage for HRS cell survival [1]. Indeed, aberrant expression of both NOTCH1 and GATA3 was suggested to play important role in cHL tumorigenesis [4, 5]. Moreover, others and we have shown that EBF1, PU.1, and FOXO1 act as tumor suppressors in cHL [6–8]. The role of TCF3 repression in cHL lymphomagenesis has not been addressed so far, but there are several lines of evidences indicating that it may act as a tumor suppressor. Contribution of TCF3 inactivation in oncogenesis have been described in other tumor entities [9–12]. Moreover, recent genome-wide association studies revealed that genetic abnormalities in the *TCF3* locus is associated with an increased risk of cHL [13]. In this study we show that reactivation of TCF3 causes cell death and cell cycle arrest in cHL cell lines. The antitumor effect of the TCF3 is accompanied by induction of proapoptotic and antiproliferative genes like *BIK, APAF1, FASLG, BOK,* and *TNFRSF10A/DR4,* and *p21.*

## RESULTS

### TCF3 negatively regulates proliferation and induces cell death

**Figure 1:**
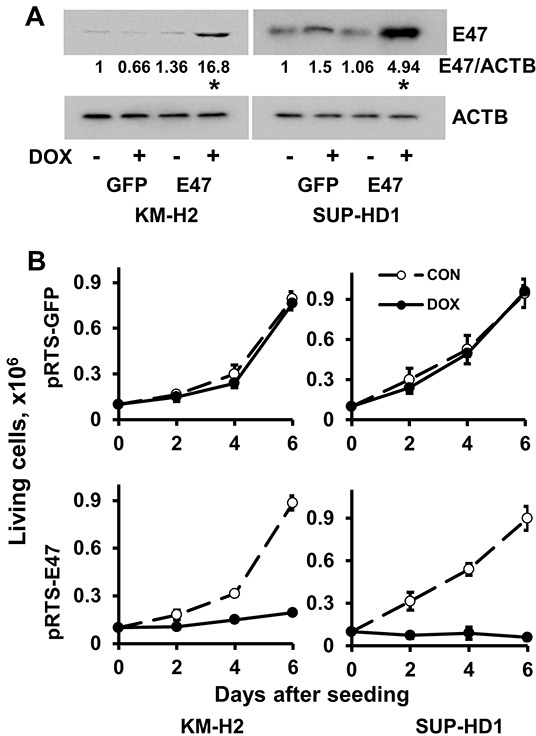
E47 induces cell death in cHL cell lines **A.** KM-H2 and SUP-HD1 cell lines conditionally expressing E47 were established using the pRTS vector system. After transfection, cells were selected with hygromycin for two weeks (KM-H2) and one week (SUP-HD1), more than 95% cells were GFP positive upon DOX treatment. Expression of E47 protein was measured by immunobloting using anti-TCF3 antibody. Anti-ACTB antibody was used as a loading control. * p<0.05 (expression levels upon DOX treatment compared with that of samples without DOX treatment) as it was assessed by double sided T-test. **B.** Cells transfected with empty pRTS vector (pRTS-GFP) or pRTS-E47 were seeded at low density (0.1 × 10^6^ cells per well in 3 ml of complete medium). DOX was added on day 0 and day 3. On day 2, day 4 and day 6, the cells were counted by flow cytometer and viable cells were discriminated using side/forward scatter parameters. **C.** The nuclear fragmentation was measured by Nicoletti method after 4 days of incubation with DOX. * p<0.05 as it was assessed by double sided T-test. **D.** The influence of E47 on cell cycle progression. Cells were seeded at density of 0.5×10^6^ per 10 ml of complete medium and treated with 0.5 μg/ml DOX. After 2 days, cell cycle distribution was measured by PI staining as described in the Materials and Methods section. Experiments were repeated three times. Results are presented as mean ± SD. * p<0.05.

**Figure 2:**
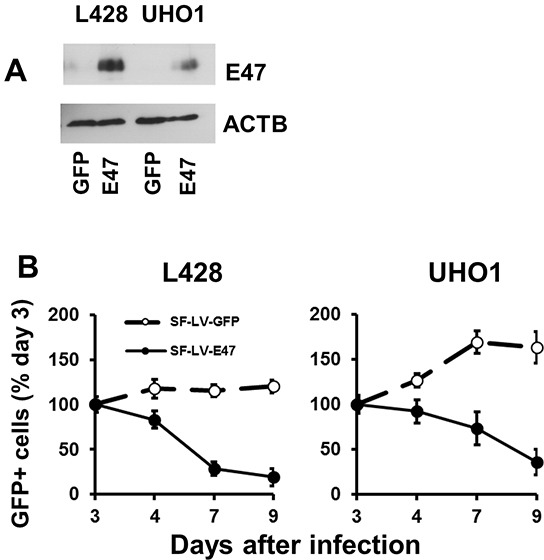
TCF3 negatively regulates proliferation E47 was over-expressed in L428 and UHO1 cells using the lentiviral SF-LV-cDNA-EGFP vector. **A.** E47 protein levels were analyzed by immunoblot. The percentage of GFP+ cells determined by flow cytometry was about 40%. Anti-ACTB antibody was used as a loading control. **B.** Percentage of GFP+ cells expressing E47 or empty vector (SF-LV) was measured at the indicated time points by flow cytometry. Percentage on the third day of infection (day 3) was defined as 100%. The data shown are representative of three independent experiments.

Since endogenous TCF3 proteins are *bona fide* inactivated by ID2 and MSC in cHL, we overexpressed E47, a TCF3 transcription variant, in KM-H2 and SUP-HD1 cell lines using doxycycline (DOX)-inducible vector pRTS containing GFP as a fluorescent marker. DOX treatment activated expression of E47 in both cell lines (Figure 1A) and decreased viable cell numbers in time-dependent manner (Figure 1B). In cell lines expressing empty vector, DOX did not influence the viability of cells (Figure 1B). To prove induction of cell death, we used Nicoletti assay. Activation of E47 led to significant nuclear fragmentation in both cell lines (Figure 1C). To assess induction of apoptosis, we used annexin-V staining. Unfortunately, due to the extremely strong GFP fluorescence of the vector that interfered with other channels, we have succeeded only in KM-H2 cells (Supplementary Figure S1A and S1B). E47 activation significantly increased proportion of annexin V positive cells in KM-H2 cells. In addition we investigated the influence of E47 expression on cell cycle progression. In both cell lines, E47 activation led to increasing proportion of cells in G0/G1 phase at the expense of cells in the S phase of the cell cycle (Figure 1D). We tried to extend our experiments on other cHL cell lines. Therefore we cloned E47 into a lentiviral vector harboring fluorescent marker (GFP). L428 and UHO1 cells were infected with lentivirus expressing E47. We validated the expression of E47 by immunoblot (Figure 2A). To assess tumor suppressor effect, we monitored the dynamics of the GFP+ population by flow cytometry (Figure 2B). Compared with cells infected with empty vector, the percentage of cells expressing E47 significantly decreased with time in both lines. Thus, we found that forcible expression of E47 induces cell death and cell cycle arrest in cHL cell lines.

### TCF3 activates proapoptotic and antiproliferative genes in cHL cell lines

To elucidate mechanisms responsible for E47 induced cell death, we used the Human Apoptosis Primer Library comprising 88 genes of the apoptosis pathway. Fifteen genes were up-regulated by more than 2-fold after E47 activation in both KM-H2 and SUP-HD1 cells (Table 1). Interestingly, no gene was downregulated by more than 2-fold in both cell lines. This is in agreement with previous observations that E47 induces far more genes than it repressed [9]. Among the 15 upregulated genes, only BIRC1 and CD40LG were anti-apoptotic and the rest 13 genes might be considered as pro-apoptotic. Eight of them belong to the class of TNF receptors and their ligands, which have not been reported to play a major role in apoptosis in cHL. The same is true for caspases 5 and 10 as well as for the BCL2-related ovarian killer BOK. The APAF1 is an essential component of the apoptosome, but it is ubiquitously expressed and its upregulation hardly can initiate apoptosis by itself. So, by exclusion we identified BCL2-interacting killer (BIK) as the most interesting candidate for further analysis. Noteworthy, *BIK* is at the top of the list of genes down-regulated in primary HRS cells over B-NHLs [14], and it was shown to be involved in B cell apoptosis [15]. *BIK* has also been reported to be upregulated upon over-expression of E47 in T cell lymphoma [9] and in a murine TCF3/E2A-deficient hematopoietic progenitor cell line [16].

**Table 1:** Genes modulated by TCF3/E2A in cHL cell lines

Official symbol	Full name	Fold of induction	
		Sup-HD1	KM-H2
APAF1	apoptotic peptidase activating factor 1	16.5±22.2	2.83±0.81
BIK	BCL2-interacting killer (apoptosis-inducing)	10.3±2.6	4.77±1.93
BIRC1	baculoviral IAP repeat-containing 1	2.31±0.06	2.21±0.09
BOK	BCL2-related ovarian killer	2.66±0.44	4.18±1.55
CASP10	Caspase 10	4.26±0.62	5.21±0.54
CASP5	Caspase 5	20.5±21.5	4.54±0.45
TNFRSF10A	tumor necrosis factor receptor superfamily, member 10A	2.55±0.34	3.62±0.4
TNFRSF11B	tumor necrosis factor receptor superfamily, member 11b	3.15±1.05	2.87±1.32
TNFRSF17	tumor necrosis factor receptor superfamily, member 17	5.04±1.5	13.7±7.76
TNFSF11	tumor necrosis factor (ligand) superfamily, member 11	5.95±1.41	2.73±1.98
TNFSF13	tumor necrosis factor (ligand) superfamily, member 13	3.8±0.63	2.14±0.14
CD40LG	CD40 ligand	7.74±1.21	5.37±2.24
FASLG	Fas ligand (TNF superfamily, member 6)	4.42±1.55	2.32±1.76
TNFSF8	tumor necrosis factor (ligand) superfamily, member 8	6.23±0.51	2.18±1.58
TRAF4	TNF receptor-associated factor 4	5.86±3.06	8.53±0.78

A total of 2 × 10^6^ KM-H2-E47 and SUP-HD1-E47 cells were seeded in 10 mL of complete culture medium and treated with or without DOX for 2 days. Then, cells were harvested, and expression of genes involved in apoptosis was analyzed using the Human Apoptosis Primer Library (Real Time Primers) Genes whose expression was changed more than 2-fold in both cell lines are shown. The data are shown as fold modulation ± SD of 3 independent experiments.

**Figure 3:**
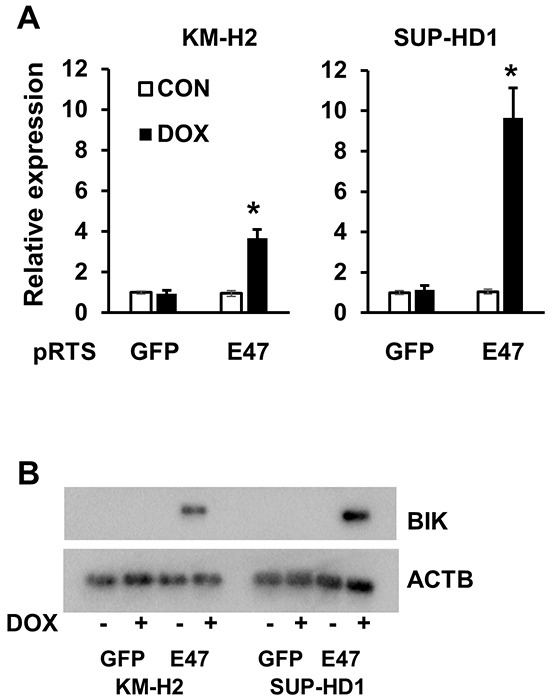
TCF3/E2A induced expression of *BIK* *BIK* is an E47 target gene in cHL cell lines. Expression of *BIK* in cHL cells expressing pRTS-GFP or pRTS-E47. Cells were treated with DOX for 2 days. RNA and protein were prepared as described in the Materials and methods section. **A.** *BIK* mRNA expression was measured by qRT-PCR. * p<0.05. **B.** BIK protein in E47-expressing cells was detected by immunobloting, in comparison with empty vector-expressing cells. The data are representative of three independent experiments with similar results.

**Figure 4:**
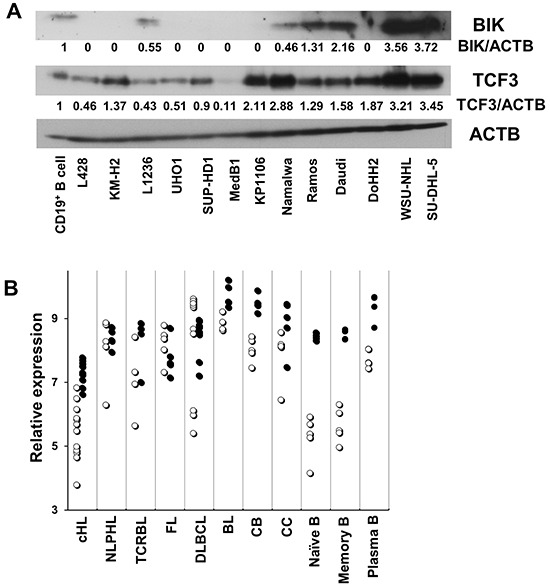
The expression of *BIK* correlates with that of *TCF3/E2A* **A.** The expression of BIK and TCF3 in various B cell lymphoma cell lines and CD19^+^ B cells was measured by immunobloting. Anti-ACTB antibody was used as a loading control. The immunoblots in (A) were quantified using ImageJ software. **B.** Published gene expression data of microdissected tumor cells including 12 cHL cases, 5 nodular lymphocyte predominant Hodgkin's lymphoma (NLPHL) cases, 4 T cell - rich B cell lymphoma (TCRBL) cases, 5 follicular lymphoma (FL) cases, 11 diffuse large B cell lymphoma (DLBCL) cases, 5 BL cases, and normal B cell subtypes (5 samples each) including centroblast (CB), centrocyte (CC), Naïve B cell (N), memory B cell (M), plasma cell (PC), were re-analyzed with help of the Genesifter software (Perkin Elmer, Seattle, WA). The data are shown as log2 of fluorescence intensity. The hollow circle (○) stands for BIK, the solid circle (•) stands for TCF3.

Consequently, we validated the upregulation of BIK by E47 in KM-H2 and SUP-HD1 cell lines using Q-RT-PCR. BIK was found upregulated in both cell lines, more pronounced in SUP-HD1 than in KM-H2 cells (over 9 and 3 folds in SUP-HD1 and KM-H2 cells, respectively) (Figure 3A). The upregulation of BIK was also detected on protein level using immunoblot (Figure 3B). Since E47 induced BIK in our cHL cell lines, we asked whether there is a correlation between expression levels of the two proteins in other tumor entities. Using immunoblot, we found that expression of BIK in most NHL cell lines was higher than in normal CD19^+^ B cells. cHL cell lines showed a virtual absence of BIK expression (Figure 4A), which is in agreement with a previous study [14]. Interestingly, MedB1 and Karpas1106, two cell lines derived from primary mediastinal B cell lymphoma (PMBL), a subtype of DLBCL that share morphological and molecular similarities with cHL, also did not express *BIK* (Figure 4A). Furthermore, we re-analyzed published gene expression profiling (GEP) data of microdissected tumor cells of 12 cHL cases, 30 B cell lymphoma cases, normal B cell subtypes (http://www.ncbi.nlm.nih.gov/geo/; GSE12453, 17.06.2013) [17]. We found that *BIK* and TCF3 were highly expressed in GC B cells, plasma cells, B-NHLs and nodular lymphocyte predominant Hodgkin's lymphoma (NLPHL). HRS cells of cHL demonstrated lowest expression levels of BIK and TCF3 (Figure 4B).

**Figure 5:**
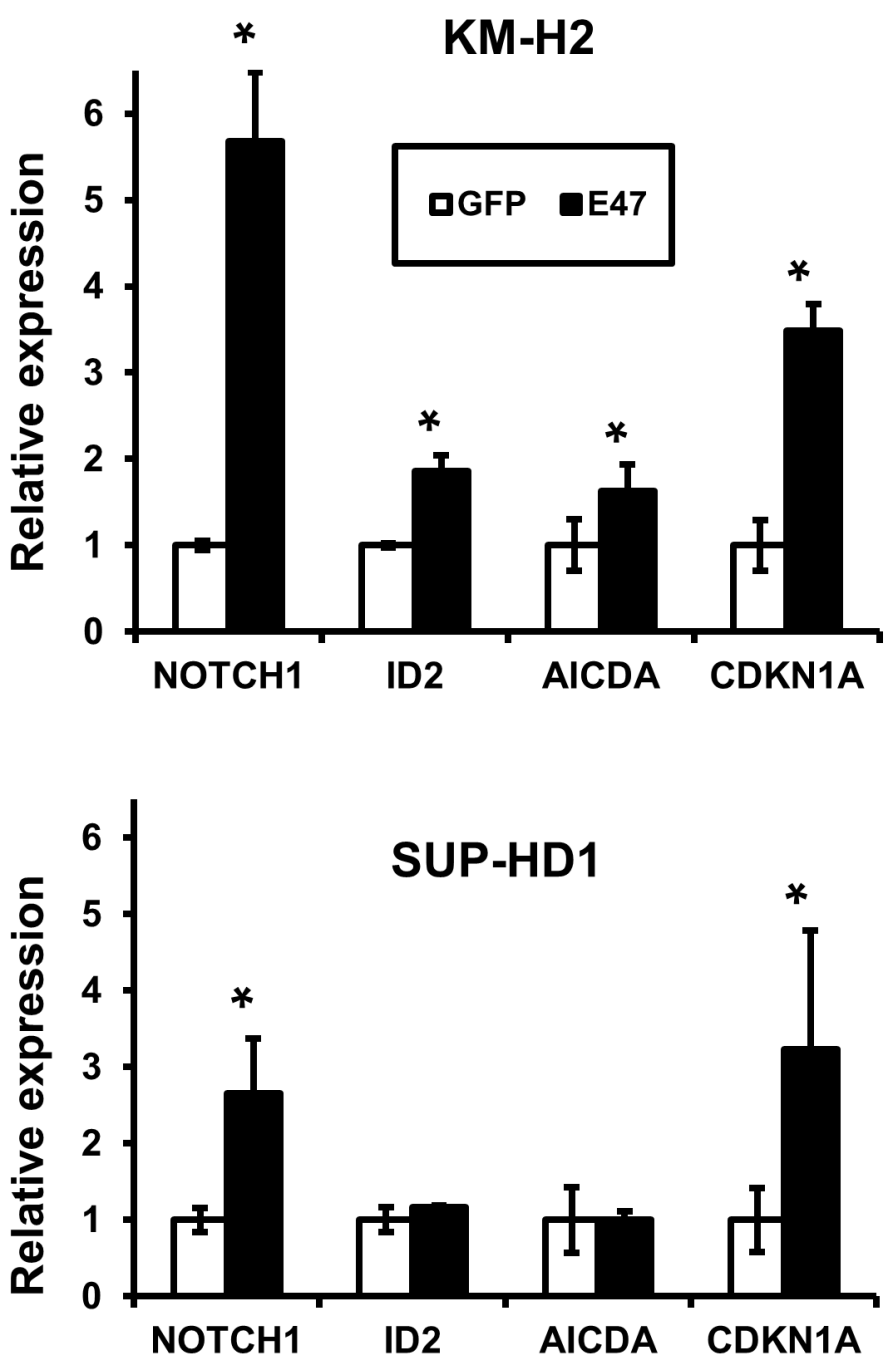
TCF3 induces expression of NOTCH1 and CDKN1A in cHL cell lines Expression of multiple genes in cHL cells expressing pRTS-GFP or pRTS-E47. Cells were treated with DOX for 2 days. RNA was prepared as described in the Materials and methods section. mRNA expression was measured by qRT-PCR. The data represent mean ± SD of three experiments. * p<0.05.

Finally we investigated whether E47 is able to reactivate known TCF3 targets like FOXO1, ABF1/MSC, POU2F2, CD19, CD79, EBF1, MYC and CDK6. We found that E47 significantly upregulated expression of NOTCH1, ID2, AICDA, and cell cycle regulator CDKN1A (Figure 5). The other genes (FOXO1, ABF1/MSC, POU2F2, CD19, CD79, EBF1, MYC, and CDK6) were not significantly modulated (data not shown). We conclude that TCF3 inactivation contributes to oncogenic program of cHL by repression of pro-apoptotic and anti-proliferative genes.

## DISCUSSION

In this study, we have shown that forced expression of *TCF3* in cHL cell lines causes apoptosis and cell cycle arrest that is associated with expression of antiproliferative and pro-apoptotic target genes. cHL is characterized by loss of the B-cell phenotype caused by repression of critical regulators of B-cell differentiation. Recently, we and other authors have shown that repression of these transcription factors including FOXO1 [6], EBF1 [8] and SPI1/PU.1 [7] has a critical impact on oncogenic program of cHL. Here we show that inactivation TCF3 also contributes to the oncogenic program of cHL. These transcription factors function in concert to regulate B cell differentiation and tumorigenesis, forming an interconnected regulatory network [18–20]. It is conceivable that deregulation of one of the regulatory units would lead to malfunction of the whole system. Particularly, given that TCF3 contributes to FOXO1 induction [20], inactivation of TCF3 might explain partially repression of FOXO1 in cHL. Our previous study showed that KLF4 repression contributes to MSC/ABF-1 activation [21]. Therefore, KLF4 inhibition might be also responsible for TCF3 inactivation.

Given that TCF3 is not only an essential regulator in B- and T- lymphocyte development, but also a tumor suppressor in T cell tumors [9, 22–25], our finding is not surprising. Similarly to cHL, in T cell lymphomas, ID proteins including ID2, block lymphocyte differentiation and promote T-cell lymphomagenesis by antagonizing TCF3 [24, 25]. In T cell lymphomas TCF3 exerts its tumor suppressing function by repression of proto-oncogenes *MYC, CDK6* and by up-regulation of cell cycling inhibitor *CDKN1A/p21*, thereby leading to cell cycle arrest [9, 26].

In regard to molecular mechanisms of TCF3 antitumor effects, in Sézary syndrome (a subtype of T cell lymphoma) derived cells, no significant cell death induction was observed after E47 overexpression, whereas, up-regulation of proapoptotic genes including *BCL2L11* and *BIK* have been seen [9, 26]. In our study, E47 induced cell death of cHL cells, which was associated with activation of multiple proapoptotic genes including BIK, APAF1, FASLG, BOK, and TNFRSF10A/DR4. Given that *BIK* is strongly expressed in GC B cells [27] and specifically repressed in HRS cells [14], this pro-apoptotic factor has attracted special attention. We have shown that the *BIK* expression significantly correlated with *TCF3* in normal B cell subtypes and B cell lymphomas. Another interesting finding was the absence of *BIK* expression in PMBL cell lines. As a distinct subtype of DLBCL, PMBL shares morphological and molecular similarities with cHL. Nevertheless, we were not able to prove contribution of BIK in the E47-induced cell death due to technical reasons. Given that TCF3 activates numerous proapoptotic genes, it is conceivable that tumor-suppressor effect does not depend on a single factor but develops as a “death by a thousand cuts” as it was supposed for IRF4 depletion in multiple myeloma [28].

BIK expression is regulated by multiple mechanisms. A recent study demonstrated that BIK expression might be a consequence of G1 cell cycle arrest in solid tumor cell lines [29]. BIK was also shown to be a p53 target gene [30] and silenced by DNA hypermethylation in some tumor entities [31, 32]. Previous studies and the present study found that BIK was upregulated by TCF3 [9, 16]. Therefore, the regulation of BIK by TCF3 might be conserved in multiple cell types. Whether this regulation is direct or indirect deserves further investigation.

We conclude that inactivation of TCF3 contributes to oncogenic program of cHL and might be critical for tumor maintenance.

## MATERIALS AND METHODS

### Cell culture and primary B cells

cHL cell lines (KM-H2, SUP-HD1, L428, UHO1, and L1236), Burkitt's lymphoma (BL) cell lines (Ramos, Namalwa, Daudi) and other B cell lymphoma cell lines (MedB1, Karpas 1106, DOHH2, WSU-NHL and SU-DHL-5) were cultured in complete RPMI 1640 medium as described earlier [21, 33]. Primary CD19^+^ B cells were isolated using microbeads (Miltenyi Biotec) as described before [21].

### Vectors and transfections

pSFI and pRTS vectors were kindly provided by D. Eick and G. W. Bornkamm [34] and were described before [18]. E47 2/5-pCS2 vector [35] was obtained from Addgene (www.addgene.org). Human E47 coding sequence was first cut out from pCS2-E47, subsequently the fragment was blunt inserted to EcoRV sites of the pSFI vector and then transferred into the pRTS-1 vector to construct the pRTS-E47 plasmid. Cells were transfected with an Amaxa Nucleofector device (Lonza, Cologne, Germany) using nucleofection buffer “V”, program Q-07 for SUP-HD1 and U-01 for KM-H2 (Lonza, Cologne, Germany). Twenty-four hours later, transfected cells were selected with 150 μg/mL Hygromycin B (InvivoGen, Toulouse, France). Efficiency of selection was monitored by FACS analysis of green fluorescent protein (GFP) expression and immunoblotting of the transgene expression 24 h after treatment with 0.5 μg/mL of Doxycycline (DOX).

To construct SF-LV-E47 vector, E47 was amplified from pCDNA3-E47 using primers: 5′-TTAACTCGAGTTAACGCCACCATGAACCAGCCGCAGAGGATG-3 and 5′-GGAGGGAGAGGGGGCTCACATGTGCCCGGCGGG-3; with restriction sites for NotI. Subsequently, E47 was introduced into the NotI restriction site of the lentiviral vector SF-LV-cDNA-EGFP (a gift of K. L. Rudolph, Leibniz Institute for Age Research, Germany [36]). Lentiviral particles were generated and cell lines were transduced as we described [37].

### Quantitative real-time PCR (qRT-PCR)

The total RNA was isolated using High Pure RNA Isolation Kit (Roche, Mannheim, Germany), first-strand cDNA with MMLV reverse transcriptase (Promega, Madison, WI). Templates amplification was done using QuantiTect SYBR Green PCR Kit (Qiagen, Hilden, Germany) on a LightCycler 480 (Roche). Primers were designed with Genscript on-line software (www.genscript.com) and synthesized by biomers.net (Ulm, Germany), sequences 5′ to 3′, sense and antisense, annealing temperature 60°C:

BIK: AGCTCCTGGAACCCCCGACC and CGC AGGGCCAATGCGTCACT [9];RPL13A: CGGACCGT GCGAGGTAT and CACCA TCCGCTTTTTCTTGTC;POU2F2: ATGGAGAAGGAAGTGATCCG and TTGA TGCGTTTCTCCTTCTG;CD19: GCAACCTGACCAT GTCATTC and TCA CAGCTGAGACCTTCCAG; CD79A: TCCTCCTCTTCCTGCTGTCT and ATC AATGATGCTGGGACCTT;EBF1: TGCTGGTCTGGAGTGA GTTG and ATGA ATCTGCCTGGTGTTCC;AICDA: CAT GGTCACCTTCAAGCTA and TTGC GTTTCCAGAAG ATTTG;CDK6: CTAGCAACCATCCCTCCATT and GGA AAGGAGCAAGAGCATTC;CDKN1A: GCAGACCAG CATGACAGATTT and GGATTAGGGCTTCCTCTTG GA;MYC: TCGGATTCTCTGCTCTCCTC and TGTT CCTCCTCAGAGTCGCT;NOTCH1: AAGATGCTCCA GCAACACAG and GGCTCTGGCAAGTCTCCTAC. Reference genes including *ACTB*, *HPRT1*, *HMBS*, and *RPL13A* were evaluated by geNorm software (http://medgen.ugent.be/~jvdesomp/genorm/). *RPL13A* was selected for calculations because it was found to be the most reliable. Human apoptosis primer library (Real Time Primers, LCC, Elkins Park, PA) was used to identify TCF3/E2A target genes as described before [21].

### Immunoblot

Immunoblot was done as described before [21]. Rabbit anti-Actin antibody (A5060, 1:15000 dilution) was from Sigma-Aldrich (Saint Louis, Missouri, USA). Rabbit antibody against human BIK protein (#4592) and TCF3/E2A (#4865) was from Cell Signaling (1:1000 dilution). Secondary antibodies included goat anti-rabbit IgG-horseradish peroxidase (HRP; sc-2004), donkey anti-goat IgG-horseradish peroxidase (HRP; sc-2020; both Santa Cruz Biotechnology). Immunoblots were quantified using ImageJ software. Anti-ACTB antibody was used as a loading control. The relative expression levels of target proteins were normalized to those of ACTB proteins.

### Cell proliferation assay and cell cycle analysis

Cell proliferation was analyzed by cell counting using a Vi-cell XR cell viability analyzer based on the trypan blue dye exclusion method (Beckman Coulter, Krefeld, Germany). Viable and dead cells were differentiated and quantified by the cell viability analyzer. Apoptosis was measured by Propidium Iodide (PI) staining and flow cytometry as described [38]. Cell cycle distribution was determined as described before [33]. Briefly, 1×10^6^ cells were fixed and stained with 70% cold ethanol and PI. DNA contents were measured by flow cytometry with a FACSCalibur flow cytometer (BD Biosciences). Data were analyzed using ModFit cell cycle analysis software (Verity Software, Topsham ME, USA).

### Statistical analysis

All experiments were independently performed three times. Data were expressed as mean ± standard deviation (SD). Student's t-test was used to determine the significance of difference between two groups, one-way ANOVA was used for multiple comparisons. All statistical analyses were carried out with SPSS13.0 software (SPSS, Chicago, IL, USA), p < 0.05 was considered statistically significant. The correlation analysis was calculated using the CORREL function in Microsoft Office Excel (Microsoft Corporation).

## SUPPLEMENTARY FIGURE


