# Supplementary material for: Repression of TCF3/E2A contributes to Hodgkin lymphomagenesis
Source: Oncotarget. 2016 May 6;7(24):36854–64. doi: 10.18632/oncotarget.9210 (PMC5095044; doi:10.18632/oncotarget.9210)
Supplement: Supplementary file 1 [file oncotarget-07-36854-s001.pdf]

SUPPLEMENTARY FIGURE

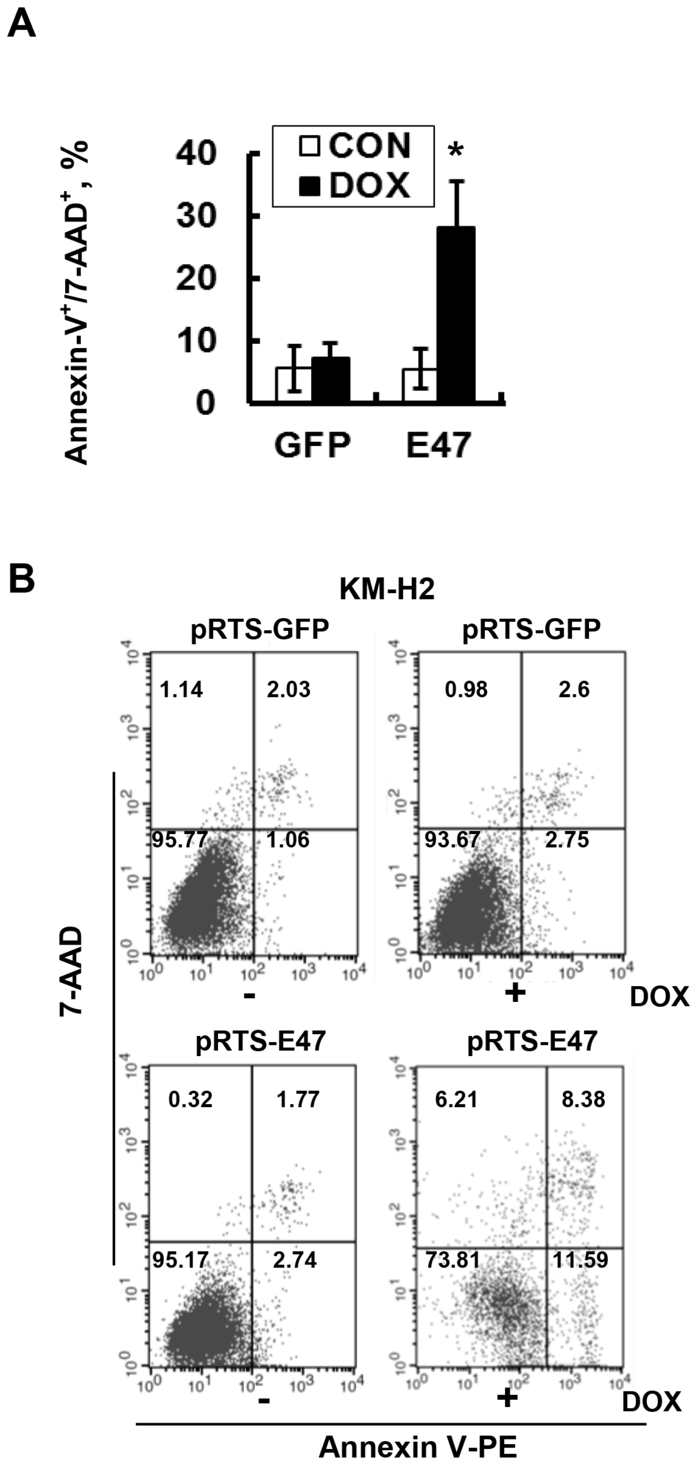

Supplementary Figure S1: A. E47 induces apoptosis in KM-H2-pRTS cell. KM-H2 cells stably expressing empty pRTS-GFP vector or pRTS-E47 were incubated in the presence of doxycycline (0.5 µg/mL) for 4 days. Cell death was measured with help of annexin V-PE/7-AAD staining. A representative one of three independent experiments yielding similar results is shown. * $p<0.05$ as it was assessed by double sided T-test. **B.** A representative fluorescence plots of annexin-V/7-AAD staining.
